# Assessment of the proarrhythmic effects of repurposed antimalarials for COVID-19 treatment using a comprehensive *in vitro* proarrhythmia assay (CiPA)

**DOI:** 10.3389/fphar.2023.1220796

**Published:** 2023-08-15

**Authors:** Seung-Hyun Yoon, Hyun-Lee Lee, Da Un Jeong, Ki Moo Lim, Seong-Jun Park, Ki-Suk Kim

**Affiliations:** ^1^ R&D Center for Advanced Pharmaceuticals and Evaluation, Korea Institute of Toxicology, Daejeon, Republic of Korea; ^2^ College of Veterinary Medicine, Research Institute of Veterinary Medicine, Chungnam National University, Daejeon, Republic of Korea; ^3^ Intelligent Human Twin Research Center, Electronics and Telecommunications Research Institute, Daejeon, Republic of Korea; ^4^ Department of IT Convergence Engineering, Kumoh National Institute of Technology, Gumi, Republic of Korea

**Keywords:** CiPA, cardiotoxicity, COVID-19, electrophysiology, antimalarials

## Abstract

Due to the outbreak of the SARS-CoV-2 virus, drug repurposing and Emergency Use Authorization have been proposed to treat the coronavirus disease 2019 (COVID-19) during the pandemic. While the efficiency of the drugs has been discussed, it was identified that certain compounds, such as chloroquine and hydroxychloroquine, cause QT interval prolongation and potential cardiotoxic effects. Drug-induced cardiotoxicity and QT prolongation may lead to life-threatening arrhythmias such as torsades de pointes (TdP), a potentially fatal arrhythmic symptom. Here, we evaluated the risk of repurposed pyronaridine or artesunate-mediated cardiac arrhythmias alone and in combination for COVID-19 treatment through *in vitro* and *in silico* investigations using the Comprehensive *in vitro* Proarrhythmia Assay (CiPA) initiative. The potential effects of each drug or in combinations on cardiac action potential (AP) and ion channels were explored using human induced pluripotent stem cell-derived cardiomyocytes (hiPSC-CMs) and Chinese hamster ovary (CHO) cells transiently expressing cardiac ion channels (Nav1.5, Cav1.2, and hERG). We also performed *in silico* computer simulation using the optimized O’Hara-Rudy human ventricular myocyte model (ORd model) to classify TdP risk. Artesunate and dihydroartemisinin (DHA), the active metabolite of artesunate, are classified as a low risk of inducing TdP based on the torsade metric score (TMS). Moreover, artesunate does not significantly affect the cardiac APs of hiPSC-CMs even at concentrations up to 100 times the maximum serum concentration (C_max_). DHA modestly prolonged at APD_90_ (10.16%) at 100 times the C_max_. When considering C_max_, pyronaridine, and the combination of both drugs (pyronaridine and artesunate) are classified as having an intermediate risk of inducing TdP. However, when considering the unbound concentration (the free fraction not bound to carrier proteins or other tissues inducing pharmacological activity), both drugs are classified as having a low risk of inducing TdP. In summary, pyronaridine, artesunate, and a combination of both drugs have been confirmed to pose a low proarrhythmogenic risk at therapeutic and supratherapeutic (up to 4 times) free C_max_. Additionally, the CiPA initiative may be suitable for regulatory use and provide novel insights for evaluating drug-induced cardiotoxicity.

## 1 Introduction

Drug-induced QT/QTc interval prolongation and cardiotoxicity are associated with arrhythmic events, such as torsade de point (TdP), and sudden cardiac death (Yap and Camm, 2003). To evaluate these risk factors, the International Council for Harmonisation of Technical Requirements for Pharmaceuticals for Human Use (ICH) has published S7B and E14 guidelines, which provide a framework for predicting cardiotoxicity assessment for drugs (Cavero and Crumb, 2005). Unfortunately, the low specificity of ICH S7B/E14 leads to the undesirable regulation of drugs that are unlikely to cause cardiac arrhythmias (Jeong et al., 2022). This is typically understood to result from focusing on only one of the many ion channels governing action potential (AP) formation (Colatsky et al., 2016; Jeong et al., 2022). To resolve this issue, the Comprehensive *in vitro* Proarrhythmia Assay (CiPA) project was proposed by the US Food and Drug Administration (FDA) and has grown into an international research project with the participation of the FDA and various researchers led by pharmaceutical companies. The CiPA project comprises four interrelated components, the first of which assesses the effects of drugs through *in vitro* experiments using ion currents participating in human ventricular AP formation (Cavero et al., 2016). The second component predicts drug-induced cardiotoxicity using an *in silico* model, the O’Hara-Rudy human ventricular myocyte model (ORd model), with results obtained from an *in vitro* ion current dataset as input (Cavero et al., 2016; Yoo et al., 2021). The *in silico* model allows the classification of each drug as having no/low, intermediate, or high proarrhythmic risk. The third component investigates the electrophysiological effects of human induced pluripotent stem cells (hiPSC-CMs), which can also be used to confirm unexpected adverse cardiac effects compared with *in vitro* multiple cardiac ion channel screening data and *in silico* prediction models (Strauss et al., 2019). The fourth component confirms *in vivo* human electrophysiological impact of drugs that may result from metabolic characteristics through human surface electrocardiography in phase I clinical trials and compares it to preclinical data (Park et al., 2019; Strauss et al., 2019). The CiPA initiative suggests a novel human-based paradigm for systematically evaluating drugs with the potential proarrhythmic risk rather than focusing on drug-induced hERG block and QT prolongation (Cavero et al., 2016; Fermini et al., 2016; Authier et al., 2017). This new paradigm can overcome the limitations of existing methods used to assess drug-induced cardiotoxicity. Moreover, the newly ICH E14/S7B Q&As incorporate CiPA assays as follow-up studies for risk evaluation best practice considerations for experimental factors (ICH E14/S7B Implementation Working Group, 2022).

Since 2019, the world has been facing a pandemic caused by coronavirus disease 2019 (COVID-19) (Touret et al., 2020; Yanagida et al., 2021); many nonclinical and clinical trials have been conducted to treat COVID-19, including the repurposing of drugs such as chloroquine (CQ), hydroxychloroquine (HCQ), and remdesivir (Yanagida et al., 2021). Drug repurposing is a useful tool for identifying new therapeutic methods (Serafin et al., 2020). Previous studies have reported that the quinoline-related antimalarial drugs CQ and HCQ block ion channels related to cardiac AP formation and can cause fatal arrhythmias associated with QT prolongation (Kinoshita et al., 2010; Borba et al., 2020; Chorin et al., 2020; Mercuro et al., 2020; Wang et al., 2020; Tleyjeh et al., 2021; Sala et al., 2022). Compliant data from the CiPA initiative have shown that using CQ and HCQ at therapeutic concentrations related to malaria or repurposing for COVID-19 is associated with the risk of QT prolongation and TdP (Delaunois et al., 2021). Those drugs inhibited mainly potassium channels, *in silico* prediction model predicted that have the potential to prolong repolarization (Thomet et al., 2021; Varshneya et al., 2021; Whittaker et al., 2021; TeBay et al., 2022). Additionally, CQ, HCQ, or azithromycin (AZM) co-administration can trigger to high arrhythmogenic risk in conditions with impaired repolarization (Sutanto and Heijman, 2020; Montnach et al., 2021). Consequently, on 15 June 2020, the FDA revoked the Emergency Use Authorization (EUA) for HCQ and CQ because they were ineffective in treating COVID-19 and could cause serious adverse cardiac events (The Food and Drug Administration, 2020a). Pyronaridine-artesunate is also an antimalarial drug that has been considered for treating COVID-19, and clinical trials are currently ongoing. Pyronaridine–artesunate is an artemisinin-based combination therapy used to treat malaria (Rueangweerayut et al., 2012). *In vitro* studies comparing the effectiveness of pyronaridine, artesunate, and HCQ against SARS-CoV-2 indicated that pyronaridine and artesunate were more effective in human lung epithelial (Calu-3) cells (Bae et al., 2020; Krishna et al., 2021). However, there is limited information on drug-induced cardiotoxicity and QT prolongation associated with repurposing for COVID-19 treatment. Therefore, it is important to determine the potential cardiotoxicity of this drug to avoid severe adverse events.

Our study aimed to perform *in vitro* and *in silico* investigations of the effects of pyronaridine, artesunate, and a combination of both drugs on cardiac electrophysiological properties and to predict arrhythmic potentials using the principles and methods of the CiPA initiative. Moreover, our study aimed to improve our understanding of cardiotoxicity and QT prolongation mechanisms, providing even greater insight into standardized nonclinical assays based on the CiPA approach.

## 2 Methods

### 2.1 Chemicals

Pyronaridine and artesunate were obtained from Sigma-Aldrich (St. Louis, MO, USA). The mixture consists of pyronaridine and artesunate in a ratio of 3:1. Dihydroartemisinin (DHA) was obtained from Selleck Chemicals (Houston, TX, USA). The drugs were formulated into a stock solution using dimethyl sulfoxide (DMSO) and stored at −20°C. On the day of the experiment, the stock solution was freshly diluted in the external solution containing 0.1% DMSO and the desired drug concentrations. All chemicals required for preparing the external/internal solutions were obtained from Sigma-Aldrich. The drugs were tested at four different concentrations, taking into account the maximum serum concentration after drug administered (C_max_) and the concentration of the drug that is not bound to plasma or tissue proteins or not uptake by red blood cells (unbound concentration) (Table 1) (The European Medicines Agency, 2012). According to The European Medicines Agency (2012), therapeutic Cmax of pyronaridine in humans is 726 ng/mL (800 nM), and unbound concentration is 12 ng/mL (13.2 nM). Pyronaridine exhibited high plasma protein binding in various organisms, including humans, dogs, rats, and rabbits ranging from 92% to 96%, and it showed preferentially associated with red blood cells. The therapeutic Cmax of artesunate in humans is 0.3 μg/mL (780 nM). The Cmax of DHA, the active metabolite of artesunate, in humans is 1.2 μg/mL (4,200 nM).

**TABLE 1:** Test concentrations of drugs used in the electrophysiological recordings (in nM).

Cardiac ion channel recordings							
Drug			C_max_	Conc.1	Conc.2	Conc.3	Conc.4
Pyronaridine			800	800	1,600	2,400	3,200
Artesunate			780	780	1,560	2,340	3,120
Dihydroartemisinin (DHA)			4,200	4,200	8,400	12,600	16,800
Mixture (3:1)	Pyronaridine		800	800	1,600	2,400	3,200
	Artesunate		780	600	1,200	1,800	2,400
Drug			Unbound concentration	Conc.1	Conc.2	Conc.3	Conc.4
Pyronaridine			13.2	13.2	26.4	39.6	52.8
Mixture (3:1)	Pyronaridine		13.2	13.2	26.4	39.6	52.8
	Artesunate		N/A	10.4	20.8	31.2	41.6
AP recordings							
Drug			C_max_	Conc.1	Conc.2	Conc.3	Conc.4
Artesunate			780	780	7,800	23,400	78,000
Dihydroartemisinin (DHA)			4,200	4,200	42,000	126,000	420,000
Drug			Unbound concentration	Conc.1	Conc.2	Conc.3	Conc.4
Pyronaridine			13.2	13.2	132	396	1,320
Mixture (3:1)		Pyronaridine	13.2	13.2	132	396	1,320
		Artesunate	N/A	10.4	104	312	1,040

### 2.2 Cell preparation

To investigate the effect of drugs on multiple cardiac ion currents (peak Nav1.5, late Nav1.5, Cav1.2, and hERG), the hERG-CHO cell line was purchased from B’SYS GmbH (Witterswil, Switzerland), while the Nav1.5-CHO and Cav1.2-CHO cell lines were purchased from Charles River Laboratories (Cleveland, OH, USA).

The hERG-CHO cells were maintained in Dulbecco’s Modified Eagle’s Medium and Nutrient Mixture F-12 (DMEM/F12) (Gibco, Gaithersburg, MD, USA) supplemented with 10% fetal bovine serum (FBS) (Gibco), 1% penicillin/streptomycin (Gibco) and 50 μg/mL hygromycin B (Invitrogen, Carlsbad, CA, USA) in a humidified 5% CO_2_ atmosphere at 37°C. The Nav1.5-CHO cells were maintained in Ham’s F-12 medium (Gibco) supplemented with 10% FBS, 1% penicillin/streptomycin, and 0.25 mg/mL Geneticin™ Selective antibiotic (Gibco) in a humidified 5% CO_2_ atmosphere at 37°C. The Cav1.2-CHO cells were maintained in Ham’s F-12 medium supplemented with 10% tetracycline screened FBS (Takara Bio Inc., Otsu, Japan), 1% penicillin/streptomycin, 0.25 mg/mL hygromycin B, 0.25 mg/mL Geneticin™ Selective antibiotic, 0.40 mg/mL Zeocin (Invivogen, San Diego, CA, USA), and 0.01 mg/mL Blasticidin (Thermo Fisher Scientific, Waltham, MA, USA) in a humidified 5% CO_2_ atmosphere at 37°C. To induce Cav1.2 expression, 1 μg/mL tetracycline (Sigma-Aldrich) was added to selection antibiotic-free media 24 h prior to testing. Additionally, to prevent Ca^2+^-induced toxicity, 3 μM verapamil hydrochloride (Sigma-Aldrich) was included.

### 2.3 Recording of ionic currents

Conventional whole-cell voltage-clamp recordings were performed for four different currents: hERG, peak Nav1.5, late Nav1.5 and Cav1.2 currents were recorded and analyzed as recommended by the FDA (The Food and Drug Administration, 2020b). The recording pipette with a tip resistance of 3–5 MΩ was filled with internal solutions. The external solution for peak Nav1.5 consisted of the following (in mM): 130 NaCl, 10 HEPES, 4 CsCl, 1 MgCl_2_*6H_2_O, 2 CaCl_2_*H_2_O, and 10 dextrose (pH 7.4 with NaOH). The internal solution for peak Nav1.5 contained the following (in mM): 130 CsCl, 7 NaCl, 1 MgCl_2_*6H_2_O, 5 HEPES, 5 EGTA, 5 Mg-ATP, and 0.4 Tris-GTP (pH 7.2 with CsOH). The same voltage protocol and solutions were used for recording the late Nav1.5 current as for as the peak Nav1.5. To induce the late Nav1.5 current, 150 nM ATX-Ⅱ (Alomone labs, Jerusalem, Israel) was added. The external solution for Cav1.2 consisted of the following (in mM): 137 NaCl, 10 HEPES, 4 KCl, 1 MgCl_2_*6H_2_O, 1.8 CaCl_2_*H_2_O, and 10 dextrose (pH 7.4 with NaOH). The internal solution for Cav1.2 contained the following (in mM): 120 Aspartic Acid, 120 CsOH, 10 CsCl, 10 HEPES, 10 EGTA, 5 Mg-ATP, and 0.4 Tris-GTP (pH 7.2 with CsOH). The external solution for hERG contained the following (in mM): 130 NaCl, 10 HEPES, 5 KCl, 1 MgCl_2_*6H_2_O, 1 CaCl_2_*H_2_O, and 12.5 dextrose (pH 7.4 with NaOH). The internal solution for hERG consisted of the following (in mM): 120 K-gluconate, 20 KCl, 10 HEPES, 5 EGTA, and 1.5 Mg-ATP (pH 7.3 with KOH). All four currents were recorded at physiological temperature (37°C ± 1°C).

### 2.4 Recording of spontaneous APs in hiPSC-CMs

For single-cell AP recordings, hiPSC-CMs (Cardiosight-S; NEXEL, Co., Ltd., Seoul, Korea) were cultured. The cells were transferred to four-well culture plates with Matrigel (Corning, NY, USA)-coated glass coverslips for AP recording. They were maintained in a culture incubator in a humidified 5% CO_2_ atmosphere at 37°C, and utilized within 7 days after thawing. The medium was changed every 2–3 days. The external solution for APs recording contained the following (in mM): 145 NaCl, 5.4 KCl, 10 HEPES, 1 MgCl_2_*6H_2_O, 1.8 CaCl_2_*H_2_O, and 5 dextrose (pH 7.4 with NaOH). The internal solution for AP recording consisted of the following (in mM): 20 KCl, 120 K-Aspartic Acid, 5 NaCl, 2 CaCl_2_*H_2_O, 5 EGTA, and 5 Mg-ATP (pH 7.25 with KOH). The maximum rate of depolarization during the upstroke of the AP (Vmax), maximum diastolic potential (MDP), AP amplitude (APA), the AP duration at 50% (APD_50_), and 90% (APD_90_) repolarization were measured when they were stable. The spontaneous contractions of hiPSC-CMs were recorded in I = 0 mode, and only cells that exhibited stable beating were used in the analysis. APs were recorded at a physiological temperature (37°C ± 1°C).

### 2.5 Hill fitting and generating samples

Hill fitting was conducted using the same method as proposed by Chang et al. (2017) with R programming language (https://github.com/FDA/CiPA/tree/Model-Validation-2018/Hill_Fitting). *In vitro* datasets obtained from the whole-cell voltage-clamp experiment were fitted and sampled with the Markov chain Monte Carlo (MCMC) model proposed by Haario et al. (2006); Laine and Tamminen (2008); Soetaert and Petzoldt (2010) to obtain Hill curves for dosage-to-response for each ion channel. The results were 2,000 samples with half-maximal inhibitory concentration (IC50) and Hill coefficients data per drug used as input for *in silico* model.

### 2.6 *In silico* model

The optimized ORd model for drug effects was used as a cellular electrophysiological *in silico* model for the human ventricular myocytes (Dutta et al., 2017), generating the AP, the sum of the potentials for all ion channels inhibited by the drugs in cardiomyocytes (O'Hara et al., 2011). The inhibited ion channel was expressed by multiplying the inhibition factor in the general ionic current equation; the inhibition factor consists of Hill coefficients $\left(h\right.$ ), IC_50_, and drug concentration ( D ) (equation 1) (Li et al., 2019).

$$Inhibition\;factor={\left[1+{\left(\frac{D}{IC50}\right)}^{h}\right]}^{-1}$$

### 2.7 Torsade metric score (TMS)

The qNet is the total amount of net charges that pass through six ion channels most affected by drugs in the cell membrane (The six ion channels are as follows: I_NaL_; the inward sodium current, I_CaL_; the L-type calcium current, I_Kr_; the rapidly activating delayed rectifier potassium current, I_Ks_; the slowly activating delayed rectifier potassium current, I_K1_; the inward rectifier potassium current, I_to_; the transient outward potassium current) during one cycle length $\left(CL\right.$ ), and is computed as equation 2 (Li et al., 2019; Yoo et al., 2021). The mean of qNet values at 1, 2, 3, and 4 multiples of C_max_ or unbound concentration for each drug, the TMS value, showed high accuracy in predicting TdP risks in the prior study of Li et al. (2019). Accordingly, this study calculated the TMS value for CiPA 12 training set drugs and derived logistic regression curves to obtain Threshold 1 and Threshold 2 for classifying TdP risk. Threshold 1 is the TMS value to separate low risk from intermediate/high risk, and Threshold 2 separates high risk from intermediate/low risk. The classifier was based on ordinal logistic regression using the lrm function from version 4.5-0 of the rms package (Chang et al., 2017). To give the logistic answer for ordinal logistic regression, the high, intermediate, and low-risk levels were categorically encoded to the numerical values of 2, 1, and 0, respectively.

$$qNet=\int_{0}^{CL}\left(INaL+ICaL+IKr+IKs+IK1+Ito\right)dt$$

### 2.8 Statistical analysis

Data analysis was performed using pCLAMP (Axon Instruments, Foster City, CA, USA), Origin 2022 (OriginLab Corp, Northampton, MA, USA), Excel (Microsoft, Redmond, WA, USA) and GraphPad Prism (GraphPad Software, San Diego, CA, USA). Statistical significance was determined using Student’s t-test. The values were presented as mean ± standard error of the mean (SEM) and were considered statistically significant when the *p*-value was less than 0.05. Statistically significant differences were assessed using the paired *t*-test at *p < 0.05, **p < 0.01 or ***p < 0.001.

## 3 Results

### 3.1 Multiple effects of drugs on cardiac ion channels

To investigate the effects of the drugs on ion channels involved in cardiac AP formation a whole-cell voltage-clamp technique was employed. In our previous studies, conducted cardiac ionic currents screening using CiPA 12 training set drugs at various multiple of C_max_ (1, 2, 3, and 4). These datasets were then utilized in the optimized ORd model, to predict drug-induced TdP risks with high accuracy. In order to enhance the accuracy of TdP prediction, it was deemed appropriate to perform cardiac ionic currents screening within the same range (1, 2, 3 and 4 multiples of C_max_ or unbound concentration) as TMS. Therefore, in this study, cardiac ionic currents screening was conducted at these specified multiples of C_max_ or unbound concentration to enable more precise prediction of TdP risks.

**FIGURE 1:**
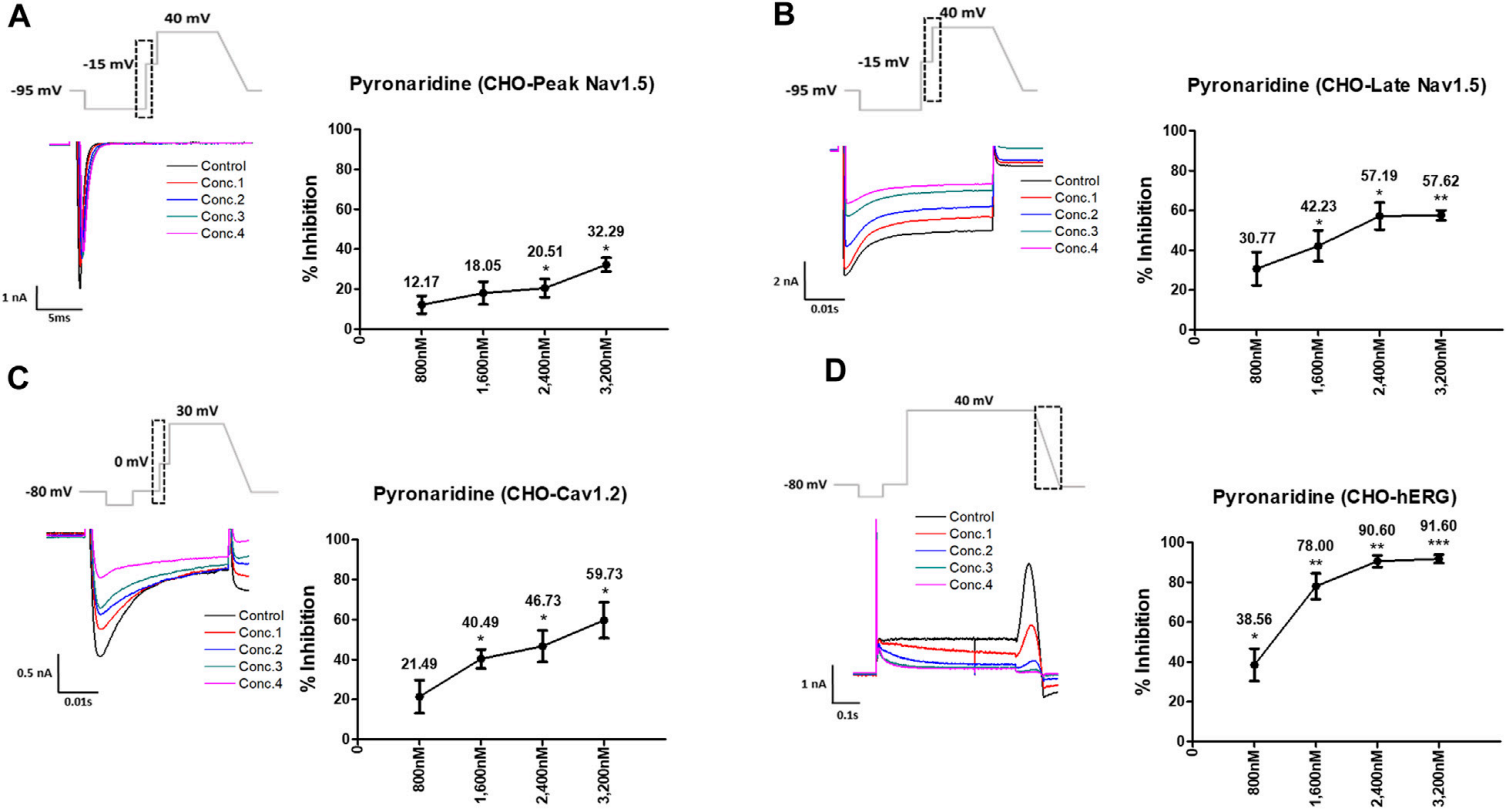
Effects of pyronaridine tested considering C_max_ on cardiac ionic currents in CHO cells. **(A–D)** Representative traces demonstrating of ionic currents showing the effect of pyronaridine on Peak Nav1.5 **(A)**, Late Nav1.5 **(B)**, Cav1.2 **(C)**, and hERG **(D)** at concentrations of 800, 1,600, 2,400, and 3,200 nM, respectively (left), and the concentration-response relationship for each ionic current (right). Data are presented as mean ± standard error of the mean (SEM) and analyzed using Student’s t-test. *p < 0.05, **p < 0.01 or ***p < 0.001 compared to control (*n* = 3).

**FIGURE 2:**
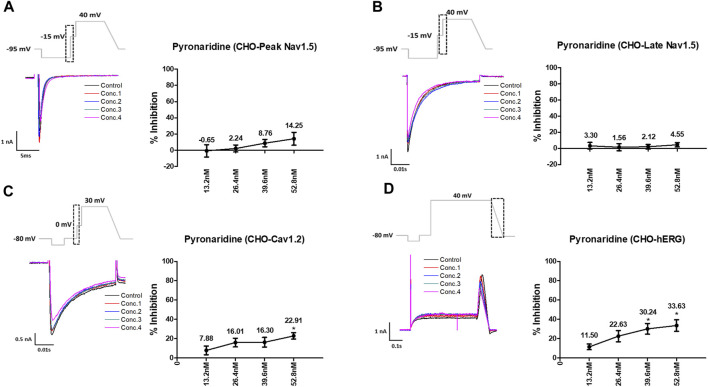
Effects of pyronaridine tested considering unbound concentration on cardiac ionic currents in CHO cells. **(A–D)** Representative traces of ionic currents demonstrating the effect of pyronaridine on Peak Nav1.5 **(A)**, Late Nav1.5 **(B)**, Cav1.2 **(C)**, and hERG **(D)** at concentrations of 13.2, 26.4, 39.6, and 52.8 nM, respectively (left), and the concentration-response relationship for each ionic current (right). Data are presented as mean ± standard error of the mean (SEM) and analyzed using Student’s t-test. *p < 0.05, **p < 0.01 or ***p < 0.001 compared to control (*n* = 3).

Pyronaridine was tested at four different concentrations (C_max_ and 2, 3, and 4 multiples of C_max_) (Figure 1). Peak Nav1.5 currents were measured as the peak inward current at the −15 mV step, and late Nav1.5 currents were measured as the inward current at the end of the −15 mV step (Figures 1A, B). Pyronaridine decreased the peak Nav1.5 at 800, 1,600, 2,400, and 3,200 nM by 12.17, 18.05, 20.51, and 32.29%, respectively. In addition, pyronaridine concentration-dependently decreased Late Nav1.5. The Cav1.2 currents were recorded as the peak inward current at 0 mV step and significantly decreased at 1,600, 2,400, and 3,200 nM by 40.49, 46.73, and 59.73%, respectively (Figure 1C). The hERG currents were measured as peak outward currents during the ramp-down phase. Pyronaridine decreased the cardiac hERG currents by 38.56, 78.00, 90.60, and 91.60% at 800, 1,600, 2,400, and 3,200 nM, respectively (Figure 1D). Pyronaridine significantly decreased the peak Nav1.5, late Nav1.5, and Cav1.2 currents; however, its sensitivity to three currents was less than that of the hERG currents. In addition, pyronaridine was tested at unbound concentrations and 2, 3, and 4 multiples of unbound concentrations (Figure 2). Pyronaridine did not significantly affect the peak Nav1.5 and late Nav1.5 currents (Figures 2A, B). The Cav1.2 currents decreased at 52.8 nM by 22.91% (Figure 2C), and the hERG currents decreased at 39.6 and 52.8 nM by 30.24% and 33.63%, respectively (Figure 2D).

**FIGURE 3:**
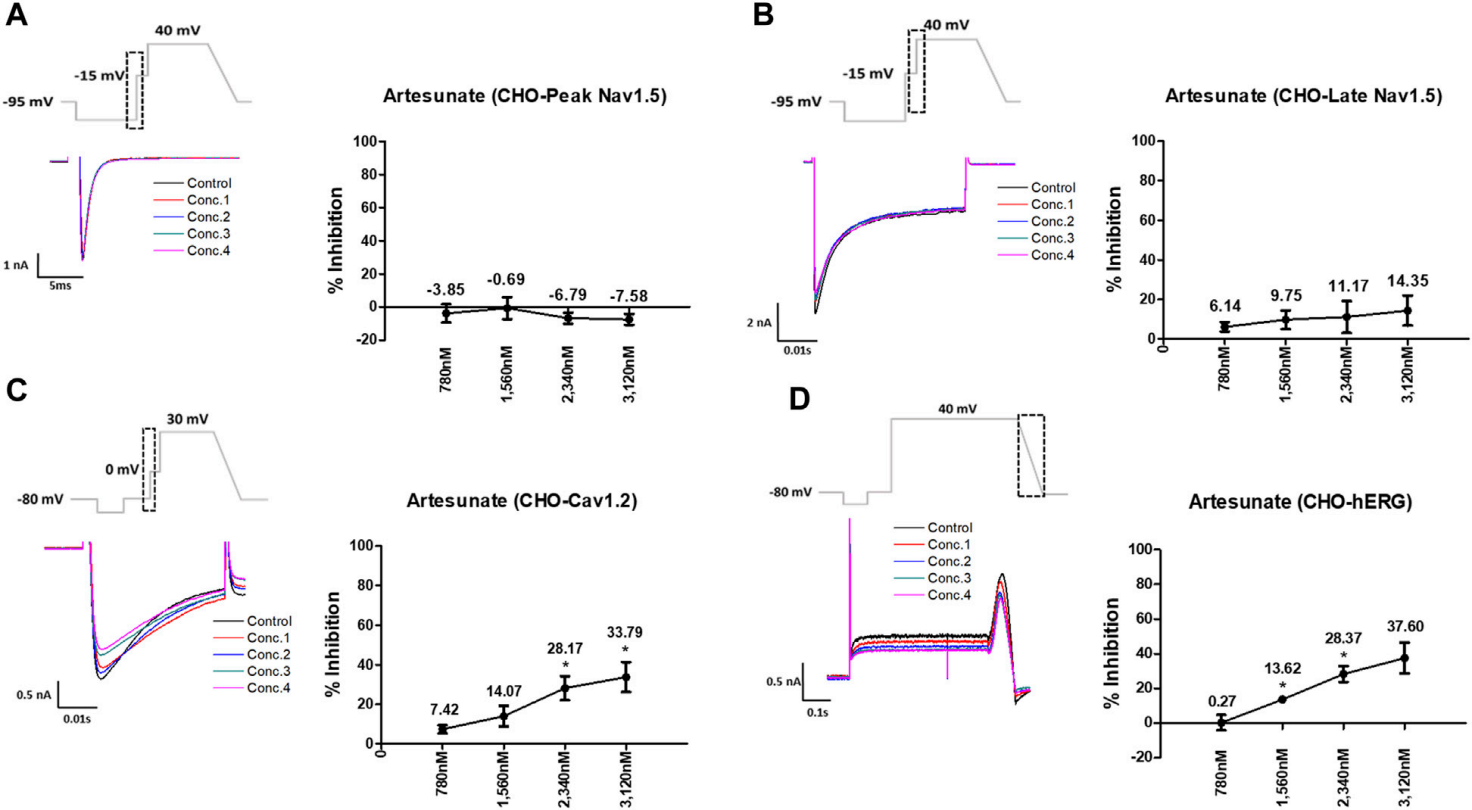
Effects of artesunate tested considering C_max_ on cardiac ionic currents in CHO cells. **(A–D)** Representative traces of ionic currents demonstrating the effect of artesunate on Peak Nav1.5 **(A)**, Late Nav1.5 **(B)**, Cav1.2 **(C)**, and hERG **(D)** at concentrations of 780, 1,560, 2,340, and 3,120 nM, respectively (left) and the concentration-response relationship for each ionic current (right). Data are presented as mean ± standard error of the mean (SEM) and analyzed using Student’s t-test. *p < 0.05, **p < 0.01 or ***p < 0.001 compared to control (*n* = 3).

**FIGURE 4:**
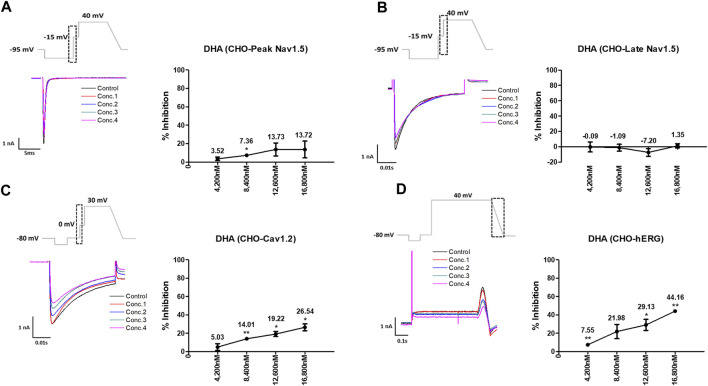
Effects of dihydroartemisinin (DHA) tested considering C_max_ on cardiac ionic currents in CHO cells. **(A–D)** Representative traces of ionic currents demonstrating the effect of DHA on Peak Nav1.5 **(A)**, Late Nav1.5 **(B)**, Cav1.2 **(C)**, and hERG **(D)** at concentrations of 780, 1,560, 2,340, and 3,120 nM, respectively (left) and the concentration-response relationship for each ionic current (right). Data are presented as mean ± standard error of the mean (SEM) and analyzed using Student’s t-test. *p < 0.05, **p < 0.01 or ***p < 0.001 compared to control (*n* = 3).

Artesunate was tested at four different concentrations (C_max_ and 2, 3, and 4 multiples of C_max_). As shown in Figure 3, artesunate did not significantly affect the peak Nav1.5 and late Nav1.5 currents (Figures 3A, B) but significantly decreased Cav1.2 currents at 2,340 and 3,120 nM by 28.17% and 33.79%, respectively (Figure 3C). Moreover, hERG currents decreased at 1,560 and 2,340 nM by 13.62% and 28.37%, respectively (Figure 3D). DHA was tested at four different concentrations (C_max_ and 2, 3, and 4 multiples of Cmax). DHA significantly decreased the peak Nav1.5 currents at 8,400 nM by 7.36% (Figure 4A). Additionally, Cav1.2 currents decreased at 8,400, 12,600, and 16,800 nM by 14.01, 19.22, and 26.54%, respectively (Figure 4C). The hERG currents significantly inhibited at 4,200, 12,600, and 16,800 nM by 7.55, 29.13, and 44.16%, respectively (Figure 4D). However, DHA did not affect the late Nav1.5 currents (Figure 4B).

**FIGURE 5:**
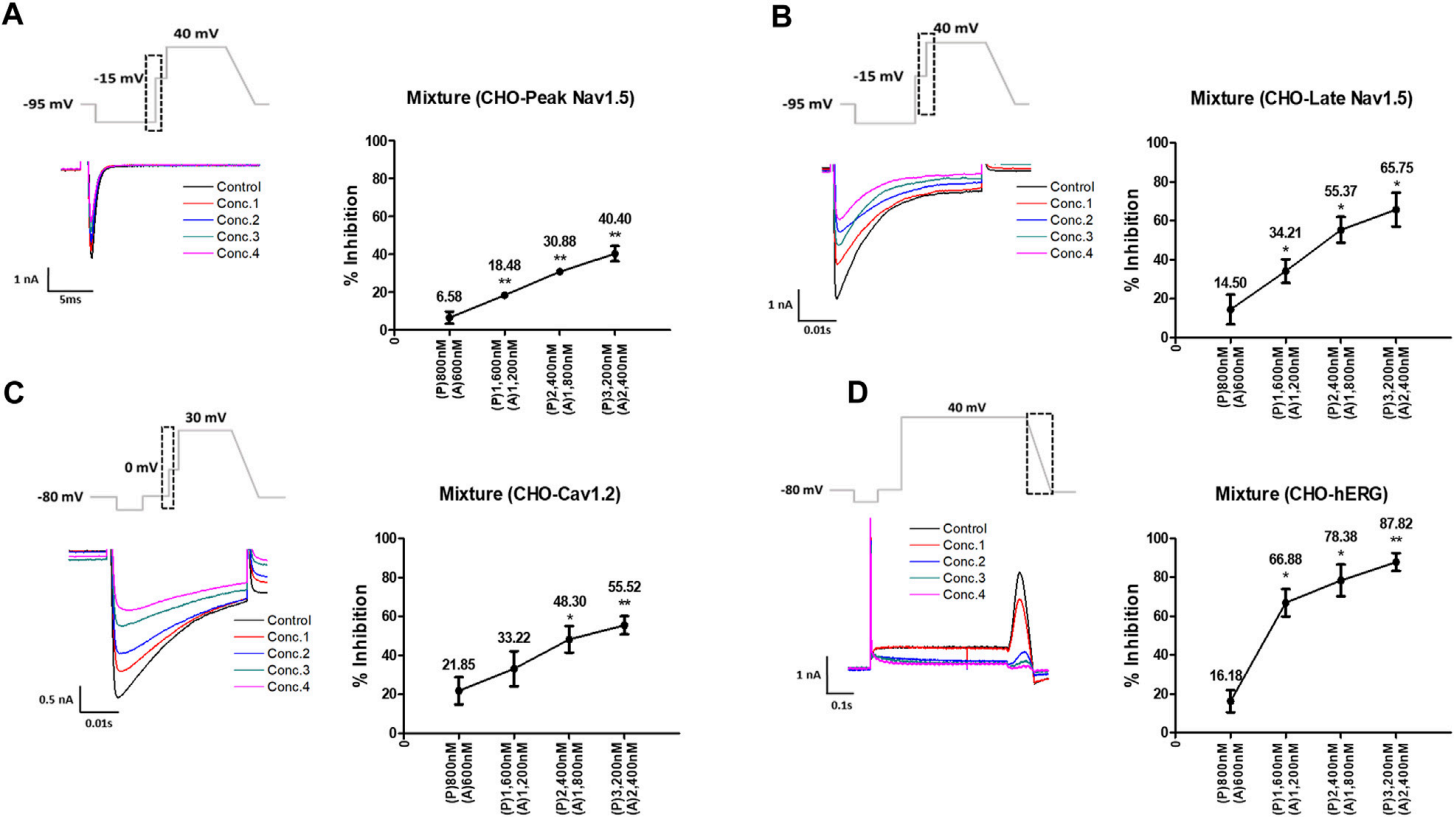
Effects of the mixture tested considering C_max_ on cardiac ionic currents in CHO cells. **(A–D)** Representative traces of ionic currents demonstrating the effect of the mixture on Peak Nav1.5 **(A)**, Late Nav1.5 **(B)**, Cav1.2 **(C)**, and hERG **(D)** with pyronaridine concentrations of 800, 1,600, 2,400, and 3,200 nM, and artesunate concentrations of 600, 1,200, 1,800, and 2,400 nM, respectively (left). Concentration-response relationship for each ionic current (right). Data are presented as mean ± standard error of the mean (SEM) and analyzed using Student’s t-test. *p < 0.05, **p < 0.01 or ***p < 0.001 compared to control (*n* = 3). P, pyronaridine; A, artesunate.

**FIGURE 6:**
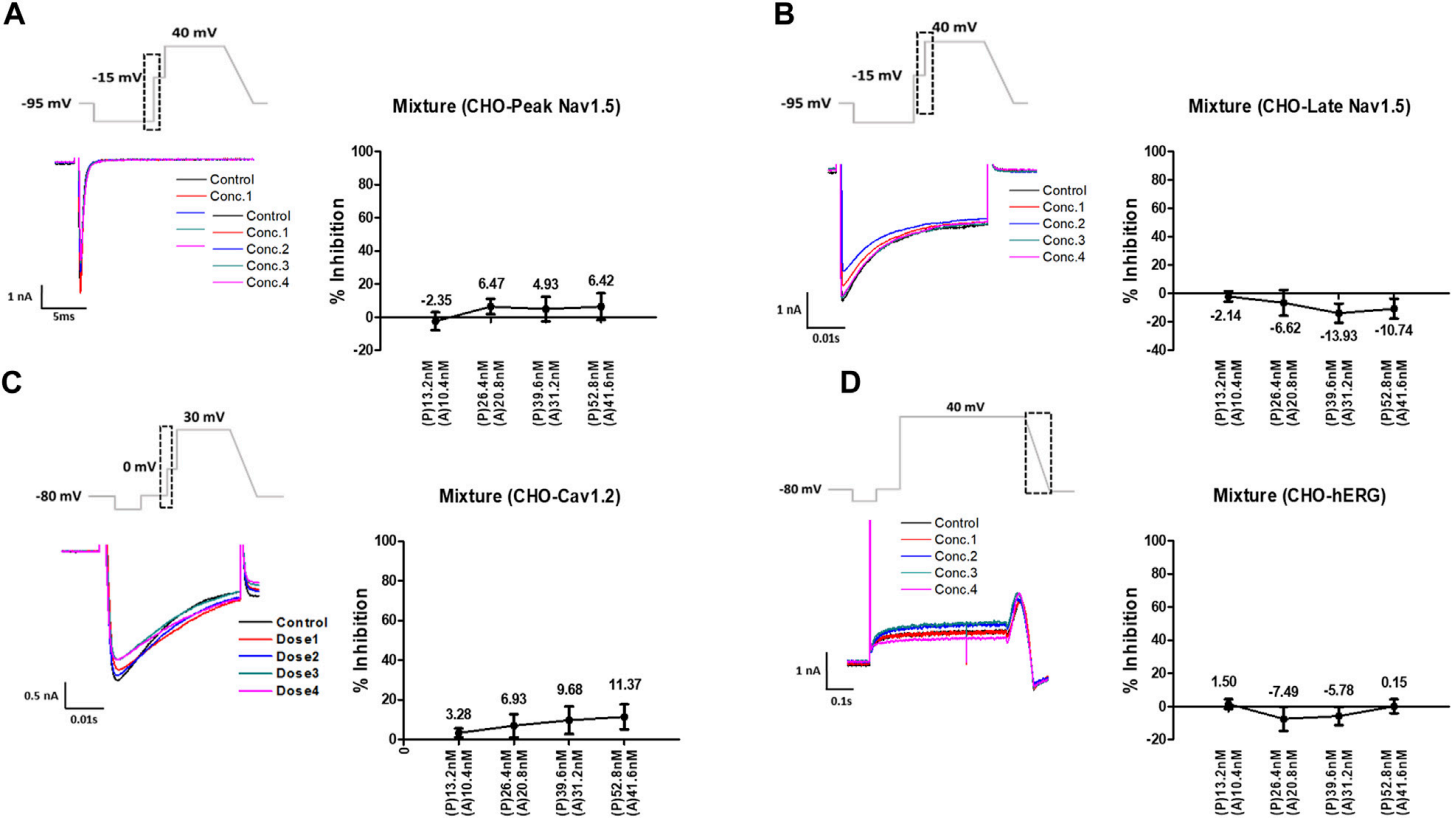
Effects of the mixture tested considering unbound concentration on cardiac ionic currents in CHO cells. **(A–D)** Representative traces of ionic currents demonstrating the effect of the mixture on Peak Nav1.5 **(A)**, Late Nav1.5 **(B)**, Cav1.2 **(C)**, and hERG **(D)** with pyronaridine concentrations of 13.2, 26.4, 39.6, and 52.8 nM and artesunate concentrations of 10.4, 20.8, 31.2, and 41.6 nM, respectively (left). Concentration-response relationship for each ionic current (right). Data are presented as mean ± standard error of the mean (SEM) and analyzed using Student’s t-test. *p < 0.05, **p < 0.01 or ***p < 0.001 compared to control (*n* = 3). P, pyronaridine; A, artesunate.

Considering the C_max_, the mixture tested decreased the four cardiac ionic currents in a concentration-dependent manner (Figure 5). Additionally, considering the unbound concentration, the mixture tested did not significantly affect the peak Nav1.5, late Nav1.5, Cav1.2, or hERG currents (Figure 6).

### 3.2 TdP risk prediction by TMS

**FIGURE 7:**
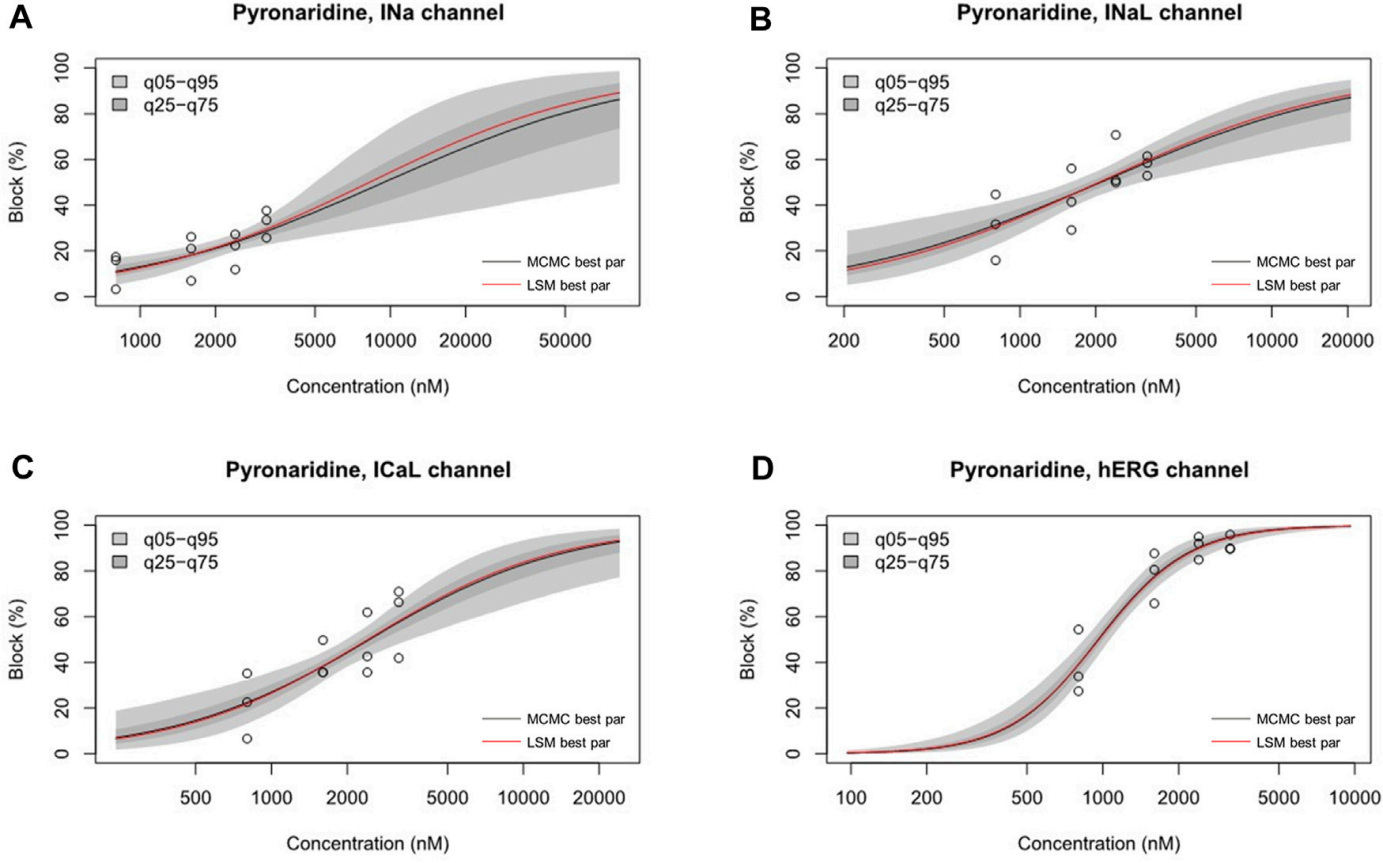
Hill curve for dosage-to-response for pyronaridine on I_Na_ **(A)**, pyronaridine on I_NaL_ **(B)**, pyronaridine on I_CaL_ **(C)**, pyronaridine on I_Kr_ **(D)**. Experimental data points for each cell by circles. 95% confidence interval of the fitting on the gray shaded area. Within each panel, the solid red line was fitted using the best parameter of the half-maximal inhibitory concentration (IC_50_) and Hill coefficient from the nonlinear least square method, whereas the solid black line was the median value from Markov chain Monte Carlo (MCMC) sampling.

**FIGURE 8:**
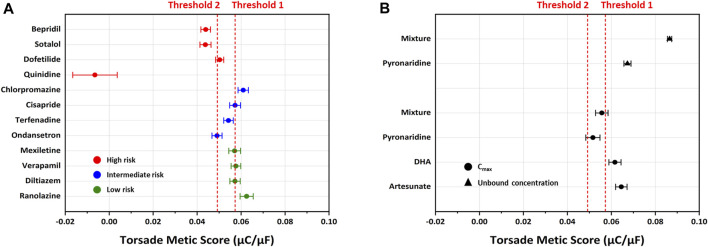
Distribution of the torsade metric score (TMS) for drugs using the optimized ORd model. Drugs are sorted based on the mean of TMS in each dataset. **(A)** Threshold 1 and threshold 2 were calculated using logistic regression to classify the TdP risk categories (red for high risk, blue for intermediate risk, green for low risk) using CiPA 12 training set drugs. Threshold 1 (separating low from intermediate/high risk) has a value of 0.0573 μC/μF, and threshold 2 (separating high from intermediate/low risk) has a value of 0.0492 μC/μF. **(B)** The drugs tested considering C_max_ are represented by solid black circle clusters, while drugs tested considering unbound concentration are represented by solid black triangle clusters.

The inhibition rates of four ion channel currents were sampled using the uncertainty quantification algorithm based on the MCMC model to generate 2,000 Hill curves. From each Hill curve, we obtained IC_50_ and Hill coefficient (Figure 7). The risk of drug-induced cardiac arrhythmias such as TdP was evaluated using a novel *in silico* biomarker, qNet, and TMS as proposed by CiPA (Dutta et al., 2017; Li et al., 2019) (Figure 8). As shown in Figure 8A, the two thresholds that classify drugs into three TdP risk categories were also calculated based on the CiPA 12 training set drugs: Threshold 1 has a value of 0.0573 μC/μF and Threshold 2 has a value of 0.0492 μC/μF. When tested considering C_max_, artesunate and DHA was classified as a low risk of inducing TdP (TMS = 0.064523 μC/μF for artesunate 0.061648 μC/μF for DHA) (Figure 8B). However, pyronaridine and the mixture were classified as having an intermediate risk of inducing TdP (TMS was 0.051608 μC/μF for pyronaridine and 0.055643 μC/μF for the mixture) (Figure 8B). In contrast, considering unbound concentration, both pyronaridine and mixture were classified as having a low risk of inducing TdP with 0.067301 μC/μF and 0.086473 μC/μF of TMS, respectively. Despite the utilization of the same drug, it was confirmed that the case tested considering the unbound concentration had a lower risk of inducing TdP than the case tested considering C_max_.

### 3.3 Effects of drugs on cardiac APs in ventricular hiPSC-CMs

**FIGURE 9:**
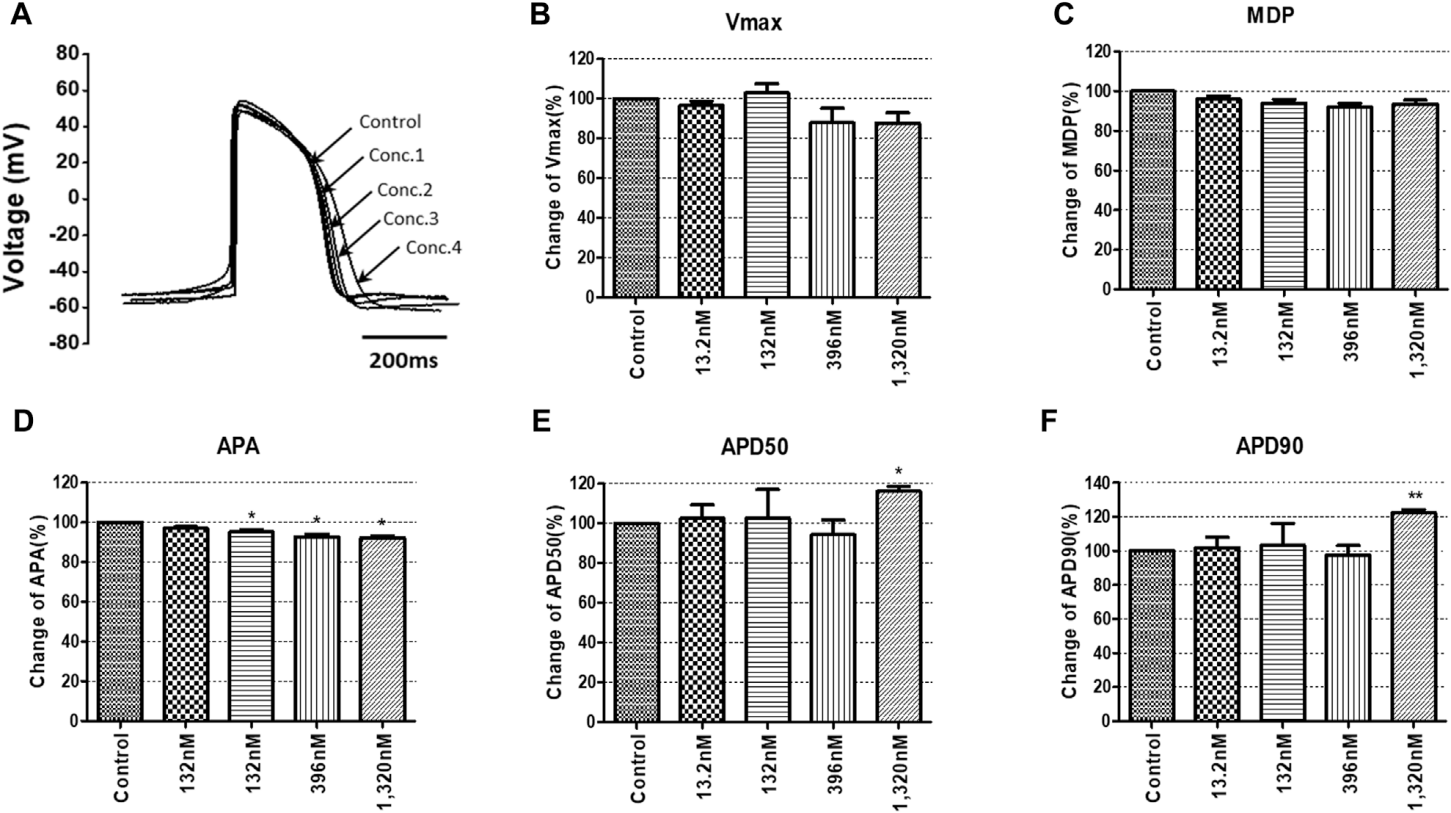
Effects of pyronaridine on action potential (AP) parameters of hiPSC-CMs. **(A)** Typical AP traces of hiPSC-CMs in the control and after exposure to 13.2, 132, 396, and 1,320 nM pyronaridine. **(B–F)** Bar graphs illustrating mean normalized values for AP parameters of hiPSC-CMs, including MDP, maximal diastolic potential; APA, AP amplitude; Vmax, maximum rate of depolarization during the upstroke of the AP; APD_50_ and APD_90_, AP duration at 50% and 90%. Data are presented as mean ± standard error of the mean (SEM) and analyzed using Student’s t-test. *p < 0.05, **p < 0.01 or ***p < 0.001 compared to control (*n* = 3).

**FIGURE 10:**
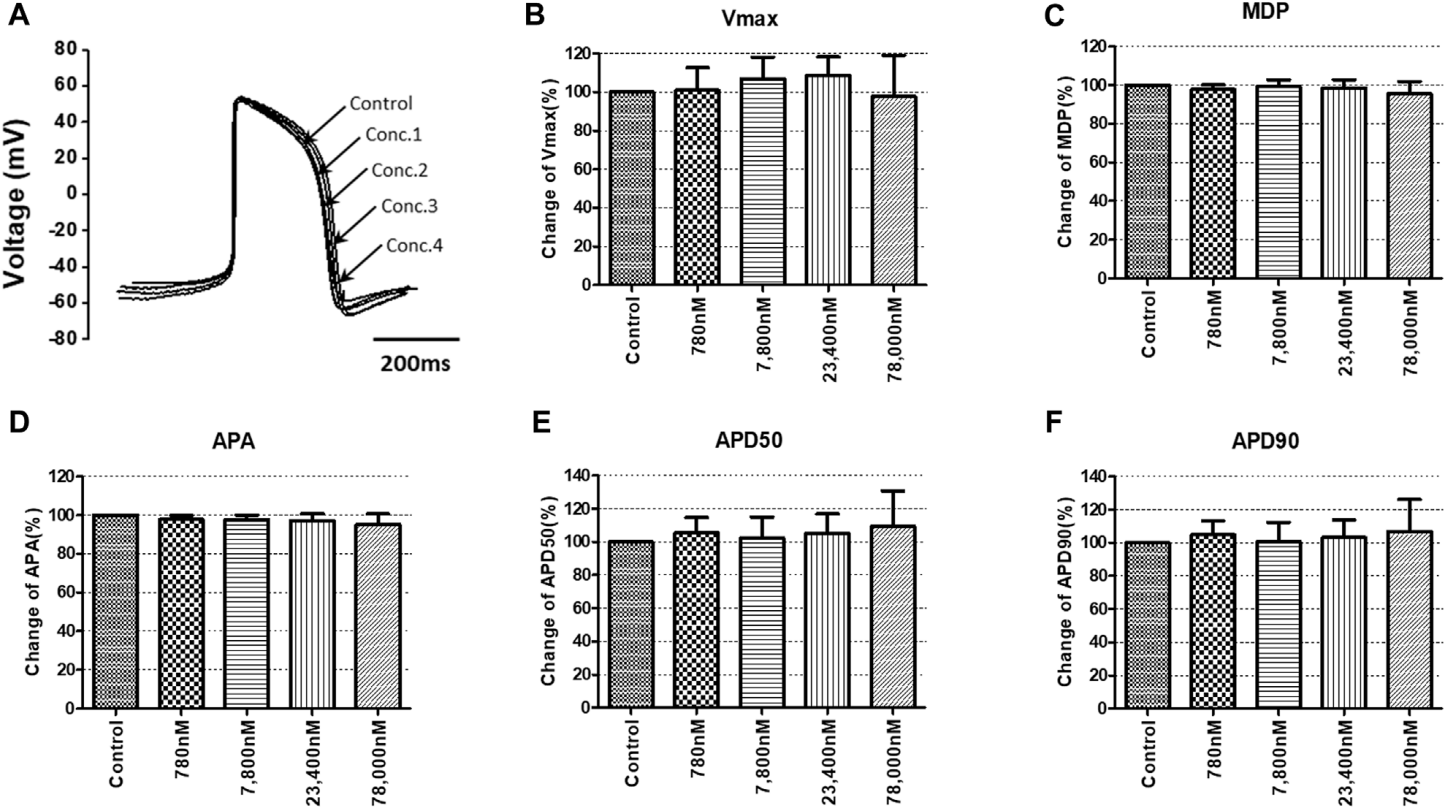
Effects of artesunate on action potential (AP) parameters of hiPSC-CMs. **(A)** Typical AP traces of hiPSC-CMs in the control and after exposure to 780, 7,800, 23,400, and 78,000 nM artesunate. **(B–F)** Bar graphs illustrating mean normalized values for AP parameters of hiPSC-CMs, including MDP, maximal diastolic potential; APA, AP amplitude; Vmax, maximum rate of depolarization during the upstroke of the AP; APD_50_ and APD_90_, AP duration at 50% and 90%. Data are presented as mean ± standard error of the mean (SEM) and analyzed using Student’s t-test. *p < 0.05, **p < 0.01 or ***p < 0.001 compared to control (*n* = 3).

**FIGURE 11:**
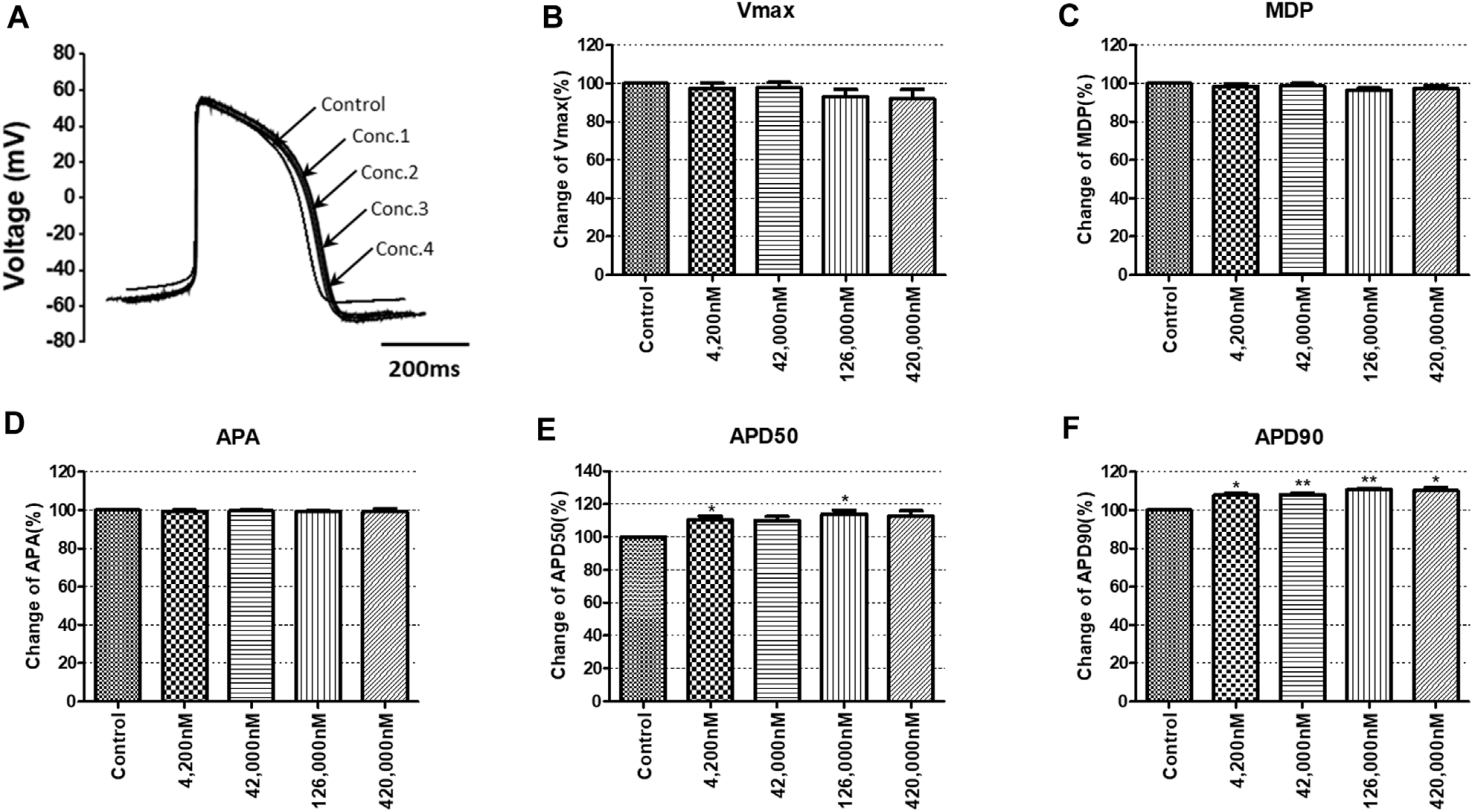
Effects of DHA on action potential (AP) parameters of hiPSC-CMs. **(A)** Typical AP traces of hiPSC-CMs in the control and after exposure to 4,200, 42,000, 126,000 and 420,000 nM DHA. **(B–F)** Bar graphs illustrating mean normalized values for AP parameters of hiPSC-CMs, including MDP, maximal diastolic potential; APA, AP amplitude; Vmax, maximum rate of depolarization during the upstroke of the AP; APD_50_ and APD_90_, AP duration at 50% and 90%. Data are presented as mean ± standard error of the mean (SEM) and analyzed using Student’s t-test. *p < 0.05, **p < 0.01 or ***p < 0.001 compared to control (*n* = 3).

**FIGURE 12:**
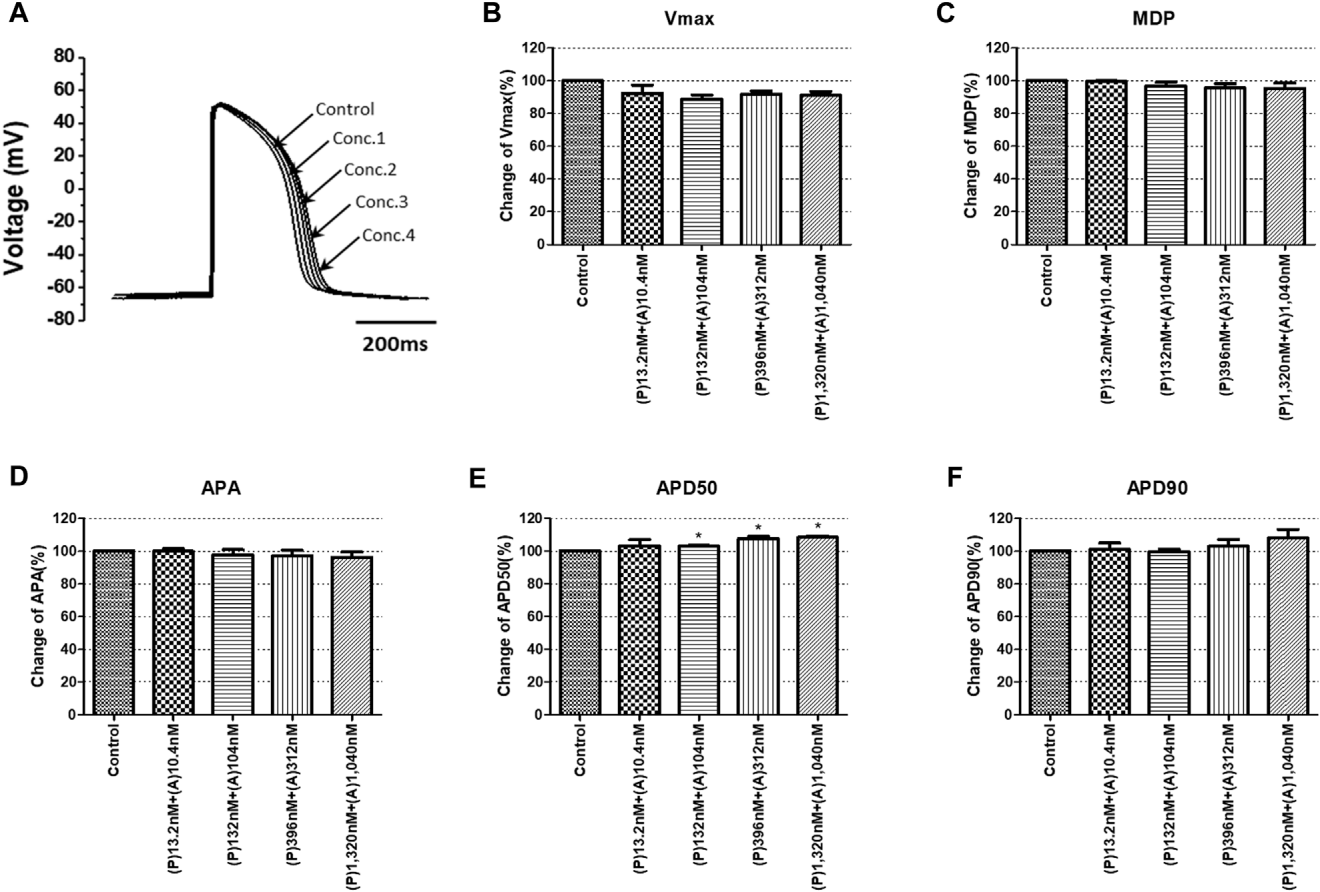
Effects of the mixture on action potential (AP) parameters of hiPSC-CMs. **(A)** Typical AP traces of hiPSC-CMs in the control and after exposure to 13.2, 132, 396, and 1,320 nM pyronaridine and 10.4, 104, 312, and 1,040 nM artesunate. **(B–F)** Bar graphs illustrating mean normalized values for AP parameters of hiPSC-CMs, including MDP, maximal diastolic potential; APA, AP amplitude; Vmax, maximum rate of depolarization during the upstroke of the AP; APD_50_ and APD_90_, AP duration at 50% and 90%. Data are presented as mean ± standard error of the mean (SEM) and analyzed using Student’s t-test. *p < 0.05, **p < 0.01 or ***p < 0.001 compared to control (*n* = 3). P, pyronaridine; A, artesunate.

To confirm the integrated electrophysiological effects of the drugs, we measured the spontaneous beating of hiPSC-CMs using the whole-cell current-clamp method. Under control conditions, the values of the AP parameters were as follows: −63.1 ± 1.6 mV for MDP, 45.8 ± 4.1 mV/ms for Vmax, 287.0 ± 17.9 ms for APD_90_, 236.1 ± 12.5 ms for APD_50_, 1.2 ± 0.03 for APD_90_/APD_50_ ratio and 113.8 ± 1.1 mV for APA (n = 9, mean ± SEM). Previous studies using hiPSC-CMs have demonstrated that ventricular-type cells exhibit certain characteristics, including a plateau phase, APD_90_ > 150 ms, and APD_90_/APD_50_ ratio of 1.07∼1.31 (Peng et al., 2010; Honda et al., 2011). The AP waveforms and parameters in our research were consistent with those reported in previous studies. For the analysis and pharmacological testing, only ventricular-type cells were included. The effects of the drugs on AP parameters were normalized with respect to the control value within each individual cell. These normalized values were then presented as bar graphs, depicting the changes in AP shape induced by the drugs. Pyronaridine was tested at four different concentrations, considering its unbound concentration. Applying 1,320 nM pyronaridine induced significant changes in APs; APD_50_ and APD_90_ increased by 16.07% and 22.34%, respectively, compared to the control (n = 3) (Figure 9). Additionally, pyronaridine significantly decreased the APA of hiPSC-CMs in a concentration-dependent manner. There were no significant changes in the Vmax or MDP with pyronaridine. Artesunate was tested at four different concentrations considering the C_max_. As shown in Figure 10, artesunate did not significantly alter the cardiac APs of hiPSC-CMs (*n* = 3). DHA was tested at four different concentrations considering the C_max_. As shown Figure 11, DHA significantly prolonged the APD_50_ of hiPSC-CMs at 4,200 and 126,000 nM by 10.26% and 13.83%, respectively. Additionally, DHA induced significant changes in APD_90_ at 4,200, 42,000, 126,000, and 420,000 nM by 7.88, 8.02, 10.74, and 10.16%, respectively. However, it did not significant changes in the Vmax, MDP, or APA. Additionally, the mixture was tested at four different concentrations considering the unbound concentration. It significantly prolonged the APD_50_ of hiPSC-CMs at Conc.2, 3, and 4 by 2.99, 7.56, and 8.32%, respectively, but did not change any other AP parameters (MDP, Vmax, APA, or APD_90_) (Figure 12).

## 4 Discussion

In our study, we utilized the CiPA initiative, a novel approach for assessing the proarrhythmic risk of drugs, to evaluate the cardiotoxicity of pyronaridine and artesunate, which were repurposed for COVID-19 treatment during the pandemic. Antimalarial drugs are frequently combined with other drugs to increase their efficacy, reduce the risk of resistant parasites, and shorten the duration of malaria treatment (Kremsner and Krishna, 2004). Among the various antimalarial drug combination therapies, artemisinin-based combination treatments (ACTs) are generally considered the most effective, offering high therapeutic efficacy and real-life safety against malaria (Dini et al., 2018). ACTs consist of an artemisinin derivative paired with a partner drug, such as pyronaridine-artesunate. It has been reported that the combination of pyronaridine and artesunate exhibits a stronger antiviral effect (Nosten and White, 2007; Kurth et al., 2011; Lane et al., 2019). These drugs have been repurposed for COVID-19 treatment, making it crucial to evaluate their cardiovascular adverse reactions, and drug-induced cardiotoxicity. The CiPA initiative proves to be an ideal method for evaluating cardiac safety during drug screening. In our study, we evaluated the drug-induced effects on cardiac arrhythmia for each drug individually and in combination, employing the CiPA initiative. Artesunate was evaluated at four different concentrations considering the C_max_ and was classified as having a low risk of inducing TdP (Figure 8B). Moreover, it had no significant impact on the cardiac APs of hiPSC-CMs (Figure 10). According to Lakhan et al. (2013), intravenous administration of artesunate did not affect the QTc interval, and no significant cardiovascular effects were observed in patients with *falciparum* malaria. Another study found that intravenous artesunate did not prolong the QT interval, even at high doses (Maude et al., 2009). DHA is the active metabolite of artemisinin compounds (e.g., artemotil, artesunate, artemether) (Woo et al., 1998). DHA was classified as a low TdP risk (Figure 8B). It significantly inhibited cardiac ion channels, but modestly prolonged at APD_90_ (10.16%) at 100 times the C_max_. According to Borsini et al. (2012), the IC_50_ on hERG current at 0.1 Hz for DHA is 7.7 ± 0.9 μM; however, it did not significantly prolong the APD_90_. Compared to our research, DHA appears that it significantly interacts with other cardiac ion channels. Furthermore, there have been no adverse cardiovascular effects reported in malaria patients treated with these drugs in clinical trials (White, 2007). Hence, artesunate and DHA have little cardiotoxicity, as indicated in previous reports.

We investigated and compared the effects of pyronaridine and the combination of both drugs on cardiac ion channels and APs at various concentrations based on the C_max_ and unbound concentration. Drugs can bind partially to various plasma or tissue proteins, and typically, it is the unbound (free) fraction of the drug that exerts pharmacological effects (Cervelli and Russ, 2010). Conversely, some drugs tend to accumulate in the myocardium and cells, prolonging their drug action (Tylutki and Polak, 2015). The heart serves as a central pharmacokinetic compartment that is easily accessible to drugs and can also be a site for their side effects (The European Medicines Agency, 2012). Therefore, it is essential to assess drugs at various concentrations, including the free fractions and higher active concentrations. As shown in Figure 8, pyronaridine and its mixtures evaluated based on C_max_ were classified as having intermediate TdP risk. However, when considering unbound concentrations, were classified as having a low risk of inducing TdP. We assessed pyronaridine and mixture at four different concentrations considering the unbound concentration for AP recordings. These significantly inhibited hERG, Nav1.5, and Cav1.2 currents, but remained relatively modest or non-existent effects on APD_90_ in hiPSC-CM (Figures 9, 12). It is expected that multiple interactions with cardiac ion channels. Similarly, verapamil is well-known hERG blocker but does not prolong the QT interval in clinical settings due to its multi-channel effects (Honda et al., 2011). While the potential extrapolation to human is not elucidated, it has been observed that pyronaridine and its metabolites are distributed in high concentrations in the liver, spleen, adrenal gland, kidney, and thyroid gland of rats (The European Medicines Agency, 2012). However, there have been no reports of high concentrations accumulating in the heart. According to the European Medicines Agency (2012), the active concentration of pyronaridine available in the systemic circulation is only 12 ng/mL, as a result of plasma protein or tissue binding. Therefore, when considering electrophysiological recordings, TdP risk prediction using TMS, and pharmacological contexts, it is expected that pyronaridine and the mixture will pose a low proarrhythmogenic risk at supratherapeutic (up to 4 times) free C_max_.

This study is the first to report the potential cardiotoxicity in electrophysiological terms of pyronaridine and artesunate, which have been repurposed for COVID-19 treatment under the new CiPA initiative (involving multiple ion channel screening, hiPSC-CMs, and a new *in silico* human-based prediction model that has finished the training phase using CiPA 12 training set drugs).

In summary, this study suggests that pyronaridine, artesunate, and their combination, repurposed for COVID-19 treatment, pose a low risk of adverse cardiac events and cardiotoxicity. Furthermore, the utilization of the CiPA initiative in drug safety assessment platforms can help identify potential cardiotoxicity and serve as an alternative to current *in vitro* and *in vivo* systems.

## Data Availability

The original contributions presented in the study are included in the article/Supplementary Material, further inquiries can be directed to the corresponding authors.

## References

[B1] Authier S. Pugsley M. K. Koerner J. E. Fermini B. Redfern W. S. Valentin J.-P. (2017). Proarrhythmia liability assessment and the comprehensive *in vitro* proarrhythmia assay (CiPA): an industry survey on current practice. J. Pharmacol. Toxicol. Methods 86, 34–43. 10.1016/j.vascn.2017.02.021 28223123

[B2] Bae J.-Y. Lee G. E. Park H. Cho J. Kim Y.-E. Lee J.-Y. (2020). Pyronaridine and artesunate are potential antiviral drugs against COVID-19 and influenza, 2020 2007 2028.225102 bioRxiv. 10.1101/2020.07.28.225102

[B3] Borba M. G. S. Val F. F. A. Sampaio V. S. Alexandre M. a. A. Melo G. C. Brito M. (2020). Effect of high vs low doses of chloroquine diphosphate as adjunctive therapy for patients hospitalized with severe acute respiratory syndrome coronavirus 2 (SARS-CoV-2) infection: a randomized clinical trial. JAMA Netw. Open. 3, e208857. 10.1001/jamanetworkopen.2020.8857 32330277 PMC12124691

[B4] Borsini F. Crumb W. Pace S. Ubben D. Wible B. Yan G.-X. (2012). *In vitro* cardiovascular effects of dihydroartemisin-piperaquine combination compared with other antimalarials. Antimicrob. Agents Chemother. 56, 3261–3270. 10.1128/aac.05688-11 22391528 PMC3370756

[B5] Cavero I. Crumb W. (2005). ICH S7B draft guideline on the non-clinical strategy for testing delayed cardiac repolarisation risk of drugs: a critical analysis. Expert Opin. Drug Saf. 4, 509–530. 10.1517/14740338.4.3.509 15934857

[B6] Cavero I. Guillon J.-M. Ballet V. Clements M. Gerbeau J.-F. Holzgrefe H. (2016). Comprehensive *in vitro* proarrhythmia assay (CiPA): pending issues for successful validation and implementation. J. Pharmacol. Toxicol. Methods 81, 21–36. 10.1016/j.vascn.2016.05.012 27233533

[B7] Cervelli M. J. Russ G. R. (2010). Chapter 73–principles of drug therapy, dosing, and prescribing in chronic kidney disease and renal replacement therapy. Philadelphia: Elsevier.

[B8] Chang K. C. Dutta S. Mirams G. R. Beattie K. A. Sheng J. Tran P. N. (2017). Uncertainty quantification reveals the importance of data variability and experimental design considerations for *in silico* proarrhythmia risk assessment. Front. Physiol. 8, 917. 10.3389/fphys.2017.00917 29209226 PMC5702340

[B9] Chorin E. Dai M. Shulman E. Wadhwani L. Bar-Cohen R. Barbhaiya C. (2020). The QT interval in patients with COVID-19 treated with hydroxychloroquine and azithromycin. Nat. Med. 26, 808–809. 10.1038/s41591-020-0888-2 32488217

[B10] Colatsky T. Fermini B. Gintant G. Pierson J. B. Sager P. Sekino Y. (2016). The comprehensive *in vitro* proarrhythmia assay (CiPA) initiative—Update on progress. J. Pharmacol. Toxicol. Methods 81, 15–20. 10.1016/j.vascn.2016.06.002 27282641

[B11] Delaunois A. Abernathy M. Anderson W. D. Beattie K. A. Chaudhary K. W. Coulot J. (2021). Applying the CiPA approach to evaluate cardiac proarrhythmia risk of some antimalarials used off‐label in the first wave of COVID‐19. Clin. Transl. Sci. 14, 1133–1146. 10.1111/cts.13011 33620150 PMC8014548

[B12] Dini S. Zaloumis S. Cao P. Price R. N. Fowkes F. J. Van Der Pluijm R. W. (2018). Investigating the efficacy of triple artemisinin-based combination therapies for treating Plasmodium falciparum malaria patients using mathematical modeling. Antimicrob. Agents Chemother. 62, e01068-18. 10.1128/AAC.01068-18 30150462 PMC6201091

[B13] Dutta S. Chang K. C. Beattie K. A. Sheng J. Tran P. N. Wu W. W. (2017). Optimization of an *in silico* cardiac cell model for proarrhythmia risk assessment. Front. Physiol. 8, 616. 10.3389/fphys.2017.00616 28878692 PMC5572155

[B14] Fermini B. Hancox J. C. Abi-Gerges N. Bridgland-Taylor M. Chaudhary K. W. Colatsky T. (2016). A new perspective in the field of cardiac safety testing through the comprehensive *in vitro* proarrhythmia assay paradigm. J. Biomol. Screen. 21, 1–11. 10.1177/1087057115594589 26170255

[B15] Haario H. Laine M. Mira A. Saksman E. (2006). Dram: efficient adaptive MCMC. Statistics Comput. 16, 339–354. 10.1007/s11222-006-9438-0

[B16] Honda M. Kiyokawa J. Tabo M. Inoue T. (2011). Electrophysiological characterization of cardiomyocytes derived from human induced pluripotent stem cells. J. Pharmacol. Sci. 117, 149–159. 10.1254/jphs.11038FP 22027094

[B17] ICH E14/S7B Implementation Working Group (2022). Questions and answers: clinical and nonclinical evaluation of QT/QTc interval prolongation and proarrhythmic potential. Available at: https://database.ich.org/sites/default/files/E14-S7B_QAs_Step4_2022_0221.pdf (Accessed June 27, 2022).

[B18] Jeong D. U. Yoo Y. Marcellinus A. Kim K. S. Lim K. M. (2022). Proarrhythmic risk assessment of drugs by d V m/dt shapes using the convolutional neural network. CPT Pharmacometrics Syst. Pharmacol. 11, 653–664. 10.1002/psp4.12803 35579100 PMC9124356

[B19] Kinoshita A. Yamada H. Kotaki H. Kimura M. (2010). Effects of anti-malarial drugs on the electrocardiographic QT interval modelled in the isolated perfused Guinea pig heart system. Malar. J. 9, 318. 10.1186/1475-2875-9-318 21067575 PMC2992072

[B20] Kremsner P. G. Krishna S. (2004). Antimalarial combinations. Lancet 364, 285–294. 10.1016/S0140-6736(04)16680-4 15262108

[B21] Krishna S. Augustin Y. Wang J. Xu C. Staines H. M. Platteeuw H. (2021). Repurposing antimalarials to tackle the COVID-19 pandemic. Trends Parasitol. 37, 8–11. 10.1016/j.pt.2020.10.003 33153922 PMC7572038

[B22] Kurth F. Bélard S. Basra A. Ramharter M. (2011). Pyronaridine–artesunate combination therapy for the treatment of malaria. Curr. Opin. Infect. Dis. 24, 564–569. 10.1097/QCO.0b013e32834cabdb 21986615

[B23] Laine M. Tamminen J. (2008). Aerosol model selection and uncertainty modelling by adaptive MCMC technique. Atmos. Chem. Phys. 8, 7697–7707. 10.5194/acp-8-7697-2008

[B24] Lakhan S. Lalit J. Hemlata S. Prashant N. (2013). Effect of artesunate on electrocardiographic QT interval in patients with plasmodium falciparum malaria. Int. J. Physiol. 1, 156–160. 10.5958/j.2320-608X.1.2.033

[B25] Lane T. R. Massey C. Comer J. E. Anantpadma M. Freundlich J. S. Davey R. A. (2019). Repurposing the antimalarial pyronaridine tetraphosphate to protect against Ebola virus infection. PLoS Negl. Trop. Dis. 13, e0007890. 10.1371/journal.pntd.0007890 31751347 PMC6894882

[B26] Li Z. Ridder B. J. Han X. Wu W. W. Sheng J. Tran P. N. (2019). Assessment of an *in silico* mechanistic model for proarrhythmia risk prediction under the CiPA initiative. Clin. Pharmacol. Ther. 105, 466–475. 10.1002/cpt.1184 30151907 PMC6492074

[B27] Maude R. J. Plewes K. Faiz M. A. Hanson J. Charunwatthana P. Lee S. J. (2009). Does artesunate prolong the electrocardiograph QT interval in patients with severe malaria? Am. J. Trop. M. Hyg. 80, 126–132. 10.4269/ajtmh.2009.08-0326 19141850 PMC2843440

[B28] Mercuro N. J. Yen C. F. Shim D. J. Maher T. R. Mccoy C. M. Zimetbaum P. J. (2020). Risk of QT interval prolongation associated with use of hydroxychloroquine with or without concomitant azithromycin among hospitalized patients testing positive for coronavirus disease 2019 (COVID-19). JAMA Cardiol. 5, 1036–1041. 10.1001/jamacardio.2020.1834 32936252 PMC7195692

[B29] Montnach J. Baro I. Charpentier F. De Waard M. Loussouarn G. (2021). Modelling sudden cardiac death risks factors in patients with coronavirus disease of 2019: the hydroxychloroquine and azithromycin case. EP Eur. 23, 1124–1133. 10.1093/europace/euab043 PMC8135857 34009333

[B30] Nosten F. White N. J. (2007). Artemisinin-based combination treatment of falciparum malaria. Am. J. Trop. Med. Hyg. 77, 181–192. 10.4269/ajtmh.2007.77.181 18165491

[B31] O'hara T. Virág L. Varró A. Rudy Y. (2011). Simulation of the undiseased human cardiac ventricular action potential: model formulation and experimental validation. PLoS Comput. Biol. 7, e1002061. 10.1371/journal.pcbi.1002061 21637795 PMC3102752

[B32] Park J. S. Jeon J. Y. Yang J. H. Kim M. G. (2019). Introduction to *in silico* model for proarrhythmic risk assessment under the CiPA initiative. Transl. Clin. Pharmacol. 27, 12–18. 10.12793/tcp.2019.27.1.12 32055576 PMC6989268

[B33] Peng S. Lacerda A. E. Kirsch G. E. Brown A. M. Bruening-Wright A. (2010). The action potential and comparative pharmacology of stem cell-derived human cardiomyocytes. J. Pharmacol. Toxicol. Methods 61, 277–286. 10.1016/j.vascn.2010.01.014 20153443

[B34] Rueangweerayut R. Phyo A. P. Uthaisin C. Poravuth Y. Binh T. Q. Tinto H. (2012). Pyronaridine–artesunate versus mefloquine plus artesunate for malaria. N. Engl. J. Med. 366, 1298–1309. 10.1056/NEJMoa1007125 22475593

[B35] Sala L. Leonov V. Mura M. Giannetti F. Khudiakov A. Moretti A. (2022). Use of hiPSC-derived cardiomyocytes to rule out proarrhythmic effects of drugs: the case of hydroxychloroquine in COVID-19. Front. Physiol. 12, e730127. 10.3389/fphys.2021.730127 PMC8829511 35153806

[B36] Serafin M. B. Bottega A. Foletto V. S. Da Rosa T. F. Hörner A. Hörner R. (2020). Drug repositioning is an alternative for the treatment of coronavirus COVID-19. Int. J. Antimicrob. Agents 55, 105969. 10.1016/j.ijantimicag.2020.105969 32278811 PMC7194941

[B37] Soetaert K. Petzoldt T. (2010). Inverse modelling, sensitivity and Monte Carlo analysis in R using package FME. J. Stat. Softw. 33, 1–28. 10.18637/jss.v033.i03 20808728

[B38] Strauss D. G. Gintant G. Li Z. Wu W. Blinova K. Vicente J. (2019). Comprehensive *in vitro* proarrhythmia assay (CiPA) update from a cardiac safety research consortium/health and environmental sciences institute/FDA meeting. Ther. Innov. Regul. Sci. 53, 519–525. 10.1177/2168479018795117 30157676

[B39] Sutanto H. Heijman J. (2020). Beta-adrenergic receptor stimulation modulates the cellular proarrhythmic effects of chloroquine and azithromycin. Front. Physiol. 11, 587709. 10.3389/fphys.2020.587709 33192602 PMC7642988

[B40] Tebay C. Mcarthur J. R. Mangala M. Kerr N. Heitmann S. Perry M. D. (2022). Pathophysiological metabolic changes associated with disease modify the proarrhythmic risk profile of drugs with potential to prolong repolarisation. Br. J. Pharmacol. 179, 2631–2646. 10.1111/bph.15757 34837219

[B55] The European Medicines Agency (2012). Pyramax. Available at: https://www.ema.europa.eu/en/documents/outside-eu-product-information/pyramax-product-information_en.pdf (Accessed Feburary 16, 2012).

[B41] The Food and Drug Administration (2020a). Fact sheet for health care providers emergency use authorization (EUA) of hydroxychloroquine sulfate supplied from the strategic national stockpile for treatment of COVID-19 in certain hospitalised patients. Available at: https://www.fda.gov/media/136537/download (Accessed June 15, 2020).

[B42] The Food and Drug Administration (2020b). Recommended voltage protocols to study drug-cardiac ion channel interactions using recombinant cell lines. Available at: https://www.fda.gov/media/131157/download (Accessed July 9, 2020).

[B43] Thomet U. Amuzescu B. Knott T. Mann S. A. Mubagwa K. Radu B. M. (2021). Assessment of proarrhythmogenic risk for chloroquine and hydroxychloroquine using the CiPA concept. Eur. J. Pharmacol. 913, 174632. 10.1016/j.ejphar.2021.174632 34785211 PMC8590616

[B44] Tleyjeh I. M. Kashour Z. Aldosary O. Riaz M. Tlayjeh H. Garbati M. A. (2021). Cardiac toxicity of chloroquine or hydroxychloroquine in patients with COVID-19: a systematic review and meta-regression analysis. Mayo Clin. Proc. Innov. Qual. Outcomes. 5, 137–150. 10.1016/j.mayocpiqo.2020.10.005 33163895 PMC7605861

[B45] Touret F. Gilles M. Barral K. Nougairède A. Van Helden J. Decroly E. (2020). *In vitro* screening of a FDA approved chemical library reveals potential inhibitors of SARS-CoV-2 replication. Sci. Rep. 10, 13093. 10.1038/s41598-020-70143-6 32753646 PMC7403393

[B46] Tylutki Z. Polak S. (2015). Plasma vs heart tissue concentration in humans–literature data analysis of drugs distribution. Biopharm. Drug Dispos. 36, 337–351. 10.1002/bdd.1944 25765563

[B47] Varshneya M. Irurzun-Arana I. Campana C. Dariolli R. Gutierrez A. Pullinger T. K. (2021). Investigational treatments for COVID-19 may increase ventricular arrhythmia risk through drug interactions. CPT Pharmacometrics Syst. Pharmacol. 10, 100–107. 10.1002/psp4.12573 33205613 PMC7753424

[B48] Wang G. Lu C.-J. Trafford A. W. Tian X. Flores H. M. Maj P. (2020). Mechanistic insights into ventricular arrhythmogenesis of hydroxychloroquine and azithromycin for the treatment of COVID-19. Biorxiv. 10.1101/2020.05.21.108605

[B49] White N. J. (2007). Cardiotoxicity of antimalarial drugs. Lancet Infect. Dis. 7, 549–558. 10.1016/S1473-3099(07)70187-1 17646028

[B50] Whittaker D. G. Capel R. A. Hendrix M. Chan X. H. S. Herring N. White N. J. (2021). Cardiac TdP risk stratification modelling of anti-infective compounds including chloroquine and hydroxychloroquine. R. Soc. Open Sci. 8, 210235. 10.1098/rsos.210235 33996135 PMC8059594

[B51] Woo S. H. Parker M. H. Ploypradith P. Northrop J. Posner G. H. (1998). Direct conversion of pyranose anomeric OH→ F→ R in the artemisinin family of antimalarial trioxanes. Tetrahedron Lett. 39, 1533–1536. 10.1016/S0040-4039(98)00132-4

[B52] Yanagida S. Satsuka A. Hayashi S. Ono A. Kanda Y. (2021). Comprehensive cardiotoxicity assessment of COVID-19 treatments using human-induced pluripotent stem cell-derived cardiomyocytes. Toxicol. Sci. 183, 227–239. 10.1093/toxsci/kfab079 34142159

[B53] Yap Y. G. Camm A. J. (2003). Drug induced QT prolongation and torsades de pointes. Heart 89, 1363–1372. 10.1136/heart.89.11.1363 14594906 PMC1767957

[B54] Yoo Y. Marcellinus A. Jeong D. U. Kim K.-S. Lim K. M. (2021). Assessment of drug Proarrhythmicity using artificial neural networks with *in silico* deterministic model outputs. Front. Physiol. 12, e761691. 10.3389/fphys.2021.761691 PMC8703011 34955882

